# Crystal structure and mol­ecular Hirshfeld surface analysis of acenaphthene derivatives obeying the chlorine–methyl exchange rule

**DOI:** 10.1107/S2056989019012428

**Published:** 2019-09-12

**Authors:** R. Sribala, S. Indhumathi, R.V. Krishnakumar, N. Srinivasan

**Affiliations:** a Department of Physics, Thiagarajar College, Madurai - 625 009, Tamilnadu, India; b School of Chemistry, Madurai Kamaraj University, Madurai - 625 021, Tamilnadu, India

**Keywords:** crystal structure, acenaphthene, supra­molecular, Hirshfeld surface, chlorine-methyl exchange

## Abstract

Instances of crystal structures that remain isomorphous in spite of some minor changes in their respective mol­ecules, such as change in a substituent atom/group, can provide insights into the factors that govern crystal packing. In this context, an accurate description of the crystal structures of an isomorphous pair that differ from each other only by a chlorine–methyl substituent, *viz.* 5′′-(2-chloro­benzyl­idene)-4′-(2-chloro­phen­yl)-1′-methyl­dispiro­[acenaphthene-1,2′-pyrrolidine-3′,3′′-piperidine]-2,4′′-dione, C_34_H_28_Cl_2_N_2_O_2_, (I), and its analogue 1′-methyl-5′′-(2-methyl­benzyl­idene)-4′-(2-methyl­phen­yl)di­spiro­[acenaphthene-1,2′-pyrrolidine-3′,3′′-piperidine]-2,4′′-dione, C_36_H_34_N_2_O_2_, (II), is presented. While there are two C—H⋯O weak inter­molecular inter­actions present in both (I) and (II), the change of substituent from chlorine to methyl has given rise to an additional weak C—H⋯O inter­molecular inter­action that is relatively stronger than the other two. However, the presence of the stronger C—H⋯O inter­action in (II) has not disrupted the validity of the chloro-methyl exchange rule. Details of the crystal structures and Hirshfeld analyses of the two compounds are presented.

## Abstract 2

The change of substituents *viz*. a chlorine atom in (I) replaced by a methyl group in (II) has not induced any differences in their respective crystal packing features, confirming the validity of the chlorine–methyl exchange rule.

## Chemical context   

The prediction of crystal structures has emerged as an exciting field involving researchers from diverse fields primarily because of its challenging complexity, which is considered analogous to that of the protein-folding problem. Attempts made in the field of crystal-structure prediction, its present status and the challenges ahead were discussed in detail in a recent article (Oganov, 2018 ▸). In this context, instances of crystal structures that remain isomorphous in spite of some minor changes in their respective mol­ecules, such as a change in a substituent atom/group, are worthy of study as they might provide some insights regarding the subtle factors that govern the crystal packing.
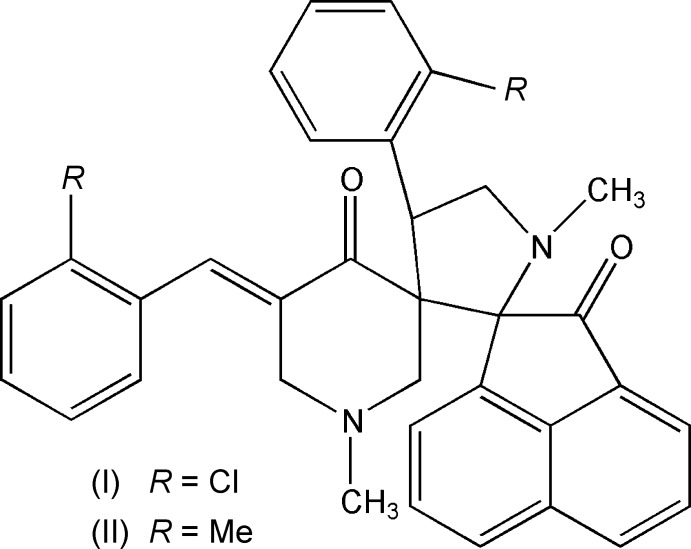


The title compounds (I) and (II) are good examples of crystal structures that obey the Cl–Me exchange rule, complying with the general conclusions arrived at in earlier studies (Jones *et al.*, 1981 ▸; Gnanaguru *et al.*, 1984 ▸; Desiraju & Sarma, 1986 ▸). In some recent studies carried out in our laboratory on mol­ecules that showcase the validity of the Cl–Me exchange rule, it has been observed that factors such as the presence of disorder and minor conformational differences have not disturbed the tendency of mol­ecules to remain as isomorphous pairs (Rajni Swamy, *et al.*, 2013 ▸; Sribala *et al.*, 2018 ▸). Inter­estingly, the validity of the Cl–Me exchange rule has also been observed in some regularly shaped planar mol­ecules (Nath & Nangia, 2012 ▸).

From a pharmacological view point, the title compounds (I) and (II) are spiro compounds that consist of a methyl­pyrrole moiety with its 2- and 3- positions as spiro carbons linked, respectively, to acenapthene and methyl pyridinone ring systems. Each of these ring systems has a variety of associated biological properties. Studies on some 4-pyridone derivatives have shown them to be potent anti­malarial agents (Bueno *et al.*, 2011 ▸) and effective in the treatment and prophylaxis of the hepatitis B virus infection (Cheng *et al.*, 2018 ▸). Acenaphthene is a pollutant known for its cytotoxicity (Jiang *et al.*, 2019 ▸) but is also useful as a dye inter­mediate. Derivatives of acenaphthene are found to exhibit anti­tumor (El-Ayaan *et al.*, 2007 ▸; Zhu *et al.*, 2008 ▸) and fungistatic properties (McDavids & Daniels, 1951 ▸). Pyrrole derivatives belong to an important class of heterocycles owing to their potential applications as anti­microbial, anti­viral, anti­malarial, anti­tubercular, anti-inflammatory and anti­cancer agents (Gholap, 2016 ▸).

## Structural commentary   

**Figure 1:**
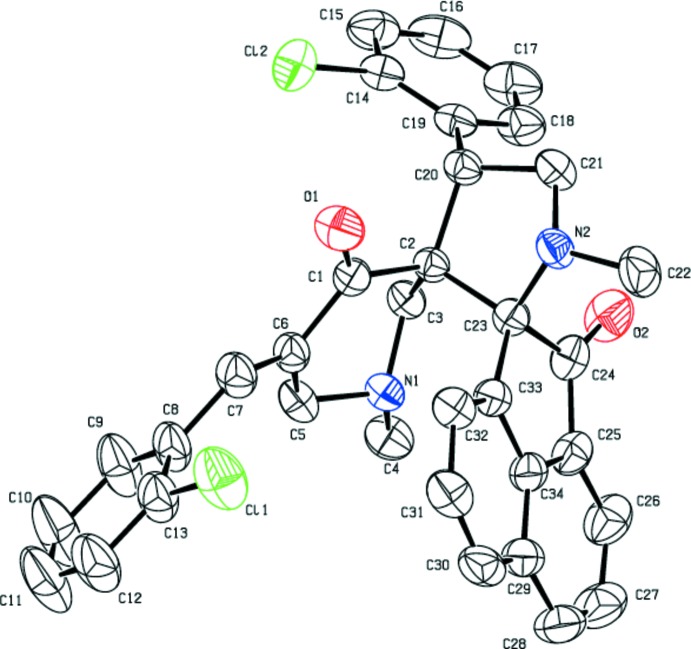
Displacement ellipsoid plot drawn at 50% probability level for (I) showing the atom-labelling scheme. H atoms have been omitted for clarity.

The mol­ecular structures of (I) and (II) (Figs. 1 ▸ and 2 ▸, respectively) differ from each other only by a chlorine atom in (I) being replaced by a methyl group in (II). This replacement has not induced any significant change in their unit-cell parameters, lattice type or space group. Similarly, there are no substantial changes in the torsion angles of the title compounds (see Tables 1 ▸ and 2 ▸), as (I) and (II) are isomorphous.

**Figure 3:**
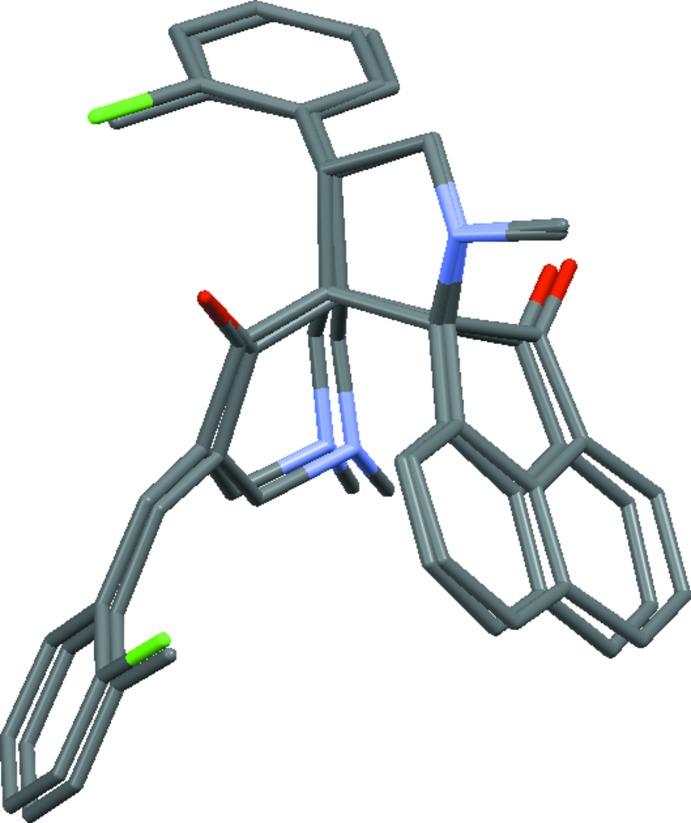
An overlay diagram depicting the superimposition of mol­ecule (I) and (II) showing no differences in the conformations.

As expected, the conformational features of both compounds are nearly identical, as shown in a overlay diagram (Fig. 3 ▸). The five-membered pyrrolo ring (N2/C21/C20/C2/C23) adopts an envelope conformation on N2 with puckering parameters *Q*(2) = 0.4011 (2) Å and φ = 180.3733 (3)° for (I), which are comparable with the values of *Q*(2) = 0.4047 (2) Å and φ = 180.3444 (3)° for (II). In both of the structures, the six-membered pyridinone ring (N1/C3/C2/C1/C6/C5) adopts a screw-boat conformation with puckering parameters *Q* = 0.5572 (16) Å, θ = 138.9 (2)°, φ = 219.8 (3)° in (I) and *Q* = 0.5603 (17) Å, θ = 137.7 (2)°, φ = 219.6 (3)° in (II). The acenaphthene ring system is planar in both (I) and (II). However, the O2 atom deviates from the mean plane of the acenaphthene ring system by 0.289 (2) Å in (I) and 0.311 (2) Å in (II), with the r.m.s. deviation of the fitted atoms being 0.043 and 0.044, respectively. This deviation is presumably due to the fact that the O2 atom is involved in two weak C—H⋯O inter­actions that are characteristic of the mol­ecular inter­action patterns of both (I) and (II).

The dihedral angle between the mean planes of the two chloro­phenyl groups in (I) is 67.66 (9)°, similarly the corresponding angle between the two methyl­phenyl groups in (II) is 66.78 (11)°. The dihedral angles between the acenaphthene ring system and the chloro­phenyl groups are 69.1 (1) and 49.4 (1)°, respectively. The corresponding angles in the methyl-substituted analogue are 72.3 (1) and 47.8 (1)°, respectively. Thus, it is clear that the minor differences observed in the conformation of the mol­ecules are insufficient to disrupt the tendency of these mol­ecules to remain isomorphous.

## Supra­molecular features   

**Figure 4:**
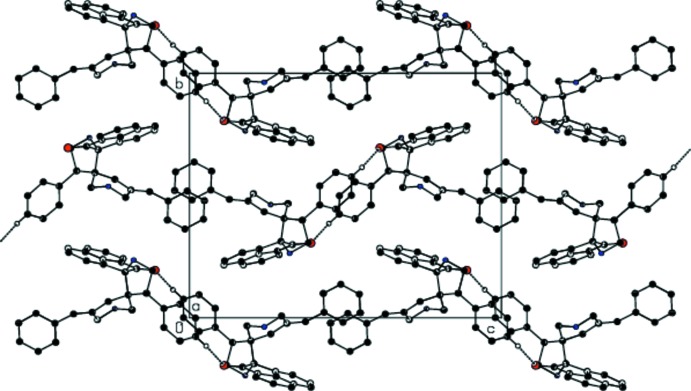
Perspective view along the *a* axis showing the weak C16—H16⋯O2 inter­molecular inter­actions between centrosymmetric pairs of mol­ecules in (I) and (II). Non-participating H atoms, methyl C atoms and Cl atoms have been omitted for clarity.

**Figure 5:**
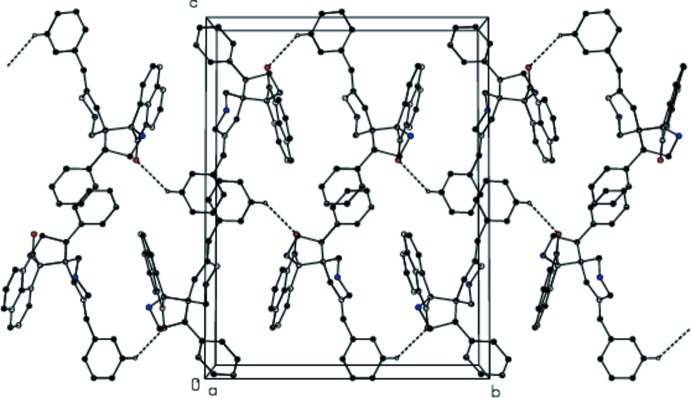
Perspective view along the *a* axis showing the weak C10—H10⋯O2 inter­molecular inter­actions between glide-related mol­ecules in (I) and (II). Non-participating H atoms, methyl C atoms and Cl atoms have been omitted for clarity.

**Figure 6:**
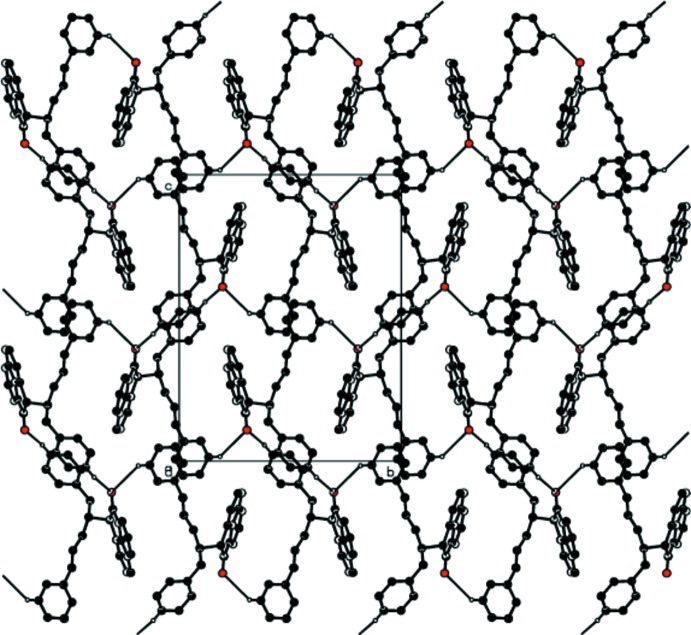
A view of the crystal structure of (I) showing the formation of 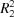
(20) graph-set motifs leading to the formation of layers formed parallel to the *bc* plane. Dashed lines indicate weak C—H⋯O inter­molecular inter­actions. H atoms not involved in the inter­actions have been omitted for clarity.

There are no classical hydrogen bonds in the structures of either (I) or (II). However, in both structures two weak C—H⋯O-type inter­molecular inter­actions, *viz*. C10—H10⋯O2 and C16—H16⋯O2, which are identical in nature and characteristic of similar fundamental mol­ecular inter­action patterns are present (Tables 3 ▸ and 4 ▸). The C16—H16⋯O2 inter­action occurs between centrosymmetric pairs (Fig. 4 ▸), leading to the formation of 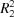
(20) graph-set motifs along the *b*-axis direction in both (I) and (II). Similarly, in both (I) and (II) the C10—H10⋯O2 inter­action links glide-related mol­ecules along the *b*-axis direction (Fig. 5 ▸). The mol­ecular aggregation pattern may be visualized as being composed of these two characteristic weak inter­actions in such a manner that centrosymmetric dimeric pairs are linked through glide-related chains of mol­ecules, forming a two-dimensional layer parallel to the *bc* plane in both structures, as shown in Figs. 6 ▸ and 7 ▸, respectively.

In (II), an additional inter­molecular inter­action is observed, *viz*. C36—H36*A*⋯O1, that is stronger than the two characteristic weak inter­molecular inter­actions and involves the replaced substituent methyl group (C36—H36*A*) as a donor and the piperidinone O1 atom as an acceptor (see Table 4 ▸). It may be concluded that the presence of this additional C—H⋯O inter­action in (II) has not disrupted the validity of the chloro–methyl exchange rule.

In addition, a weak C—H⋯π inter­action involving different donor groups and acceptor π-ring systems is present in both (I) and (II). The C5—H5*B*⋯π inter­action observed in (I) is between the C5 atom of the methyl­piperidinone ring as a donor and the C25—C29/C34 ring of the acenaphthenone system as an acceptor. Inter­estingly, a geometrically identical weak π–π inter­action about an inversion centre is observed with centroid–centroid *Cg*3⋯*Cg*3(1 − *x*, 2 − *y*, −*z*) distances of 3.7459 (2) Å in (I) and 3.8351 (2) Å in (II) with respective slippages of 1.250 and 1.367 Å where *Cg*3 is the centroid of the C14–C19 ring. The shortest Cl⋯Cl distance observed [Cl1 ⋯Cl1(−*x* + 1, −*y* + 2, −*z* + 1)] is 4.088 (1) Å and bears no structural significance.

## Database survey   

A thorough search in the Cambridge Structural Database (CSD Version 5.39, update Nov 2017; Groom *et al.*, 2016 ▸) using the main skeleton of the title compounds (having 3D coordinates with no disorder, no ions and no other errors with *R* factors less than 0.05) gave only three hits: 5′′-(4-chloro­benzyl­idene)-4′-(4-chloro­phen­yl)-1′,1′′-dimethyl-2*H*,4′′*H*-di­spiro­[ace­naphthyl­ene-1,2′-pyrrolidine-3′,3′′-piperidine]-2,4′′-dione (YIRKUG; Pandiarajan *et al.*, 2008 ▸), 5′′-benzyl­idene-1′,1′′-dimethyl-4′-phenyl-acenapthene-2-spiro-2′-pyrrolidine-3′-spiro-3′′-piperidine-1,4′′-dione (MAJHEL; Aravindan *et al.*, 2004 ▸) and 1-methyl-4-(4-methyl­phen­yl)pyrrolo-(spiro­[2.2′′]acenaphthene-1′′-one)-spiro-[3.3′]-(spiro-[5′.5′′′]-3′′′-(4-chloro­phen­yl)-4′′′-(4-methyl­phen­yl)-isoxazoline)-1′-methyl­tetra­hydro-4′(1*H*)-pyridinone (XUQFOF; Kumar, *et al.*, 2009 ▸).

## Hirshfeld surface analysis   

Hirshfeld surface (HS) analysis was used to investigate and visualize the weak inter­molecular inter­actions influential in the packing of the mol­ecules in the crystal. The visual representation of mol­ecular inter­actions on this isosurface is determined using two parameters, *viz. d*_i_ and *d*_e _, which represent the distances from a given point on the surface to the nearest atom inside and outside the surface, respectively. The normalized contact distance, *d*_norm_ is based on the values of *d*_i_ and *d*_e_.

**Figure 8:**
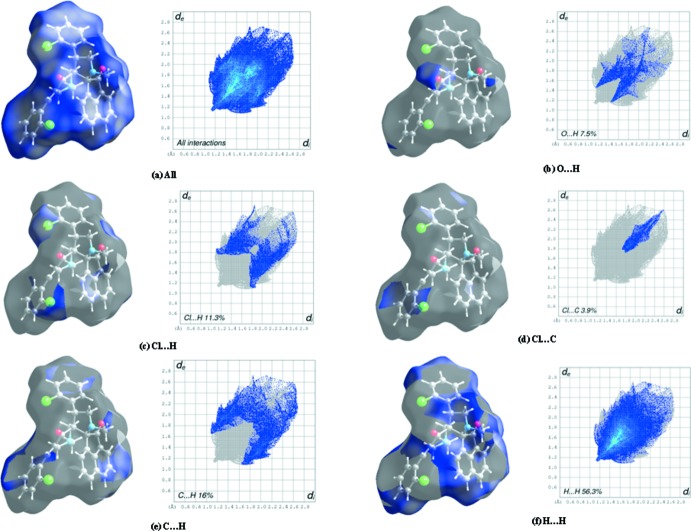
Hirshfeld surface of (I) mapped over shape-index and *d*_norm_ and decomposed fingerprint plots of the dominant inter­actions.

In the present work, the Hirshfeld surfaces (Spackman & Jayatilaka, 2009 ▸) and the associated two-dimensional fingerprint plots for title compounds (I) and (II) were generated using *CrystalExplorer3.0* (Wolff *et al.*, 2012 ▸). The Hirshfeld surfaces mapped over *d*_norm_ together with decomposed finger print plots (McKinnon *et al.*, 2007 ▸; Tan *et al.*, 2019 ▸) for (I) and (II) are presented in Figs. 8 ▸ and 9 ▸, respectively. Being isomorphic and isostructural in nature, both (I) and (II) display similar C—H⋯O inter­molecular inter­actions. The combined O⋯H and H⋯O inter­actions appear symmetrically as distinct spikes at the bottom of the fingerprint plot and contribute 7.5 and 6.9%, respectively, of the total surface in compounds (I) and (II).

The symmetrical inter­nal wing-like projections correspond to C⋯H/H⋯C contacts, which account for 16% of the HS in (I) and 19.1% in (II). The dominant contribution is from the H⋯H contacts [56.3% in (I) and 70.2% in (II)], as shown by the area occupied between the spikes. Such prominent differences may be accounted for by the presence of a Cl⋯H/H⋯Cl contact in (I) (11.3% contribution) and its absence in (II).

## Synthesis and crystallization   

For (I), a mixture of 1-methyl-3,5-bis­[(*E*)-2-chloro­phenyl­methyl­idene] tetra­hydro-4(1*H*)- pyridinone (1 mmol), acenaphthene­quinone (1 mmol) and sarcosine (1 mmol) was dissolved in methanol (15 mL) and refluxed for 30 min. After completion of the reaction, as evident from TLC, the mixture was poured into water (50 mL) and the precipitated solid was filtered and washed with water (100 mL) to obtain pure (I) as a yellow solid, (0.31 g, 98%), mp 448–449 K, *R_f_* (petroleum ether/EtOAc, 4:1) 0.40. Suitable crystals for single-crystal X-ray studies were obtained by recrystallization of the product from ethanol.

A similar procedure for (II) was adopted by dissolving a mixture of 1-methyl-3,5-bis­[(*E*)-2-methyl­phenyl­methyl­idene] tetra­hydro-4(1*H*)-pyridinone (1 mmol), acenaphthene­quinone (1 mmol) and sarcosine (1 mmol) in methanol (15 mL) to yield yellow crystals.

## Refinement   

Crystal data, data collection and structure refinement details are summarized in Table 5 ▸. C-bound H atoms were included in calculated positions and treated as riding, with C—H = 0.95–1.00 Å and *U*_iso_(H) = 1.5*U*_eq_(C) for methyl H atoms or 1.2U_eq_(C) otherwise. The H atoms of the methyl atoms C35 and C36 in (II) were refined as idealized and disordered over two positions since significant residual electron densities were noticed between the three hydrogen atoms of the respective methyl C atoms. The introduction of a disordered model for these two methyl groups had appreciable impact on the final structural parameters.

## Supplementary Material

Crystal structure: contains datablock(s) I, II. DOI: 10.1107/S2056989019012428/jj2215sup1.cif

Structure factors: contains datablock(s) I. DOI: 10.1107/S2056989019012428/jj2215Isup2.hkl

Structure factors: contains datablock(s) II. DOI: 10.1107/S2056989019012428/jj2215IIsup3.hkl

CCDC references: 1951894, 1569029

Additional supporting information:  crystallographic information; 3D view; checkCIF report

## Figures and Tables

**Figure 2:**
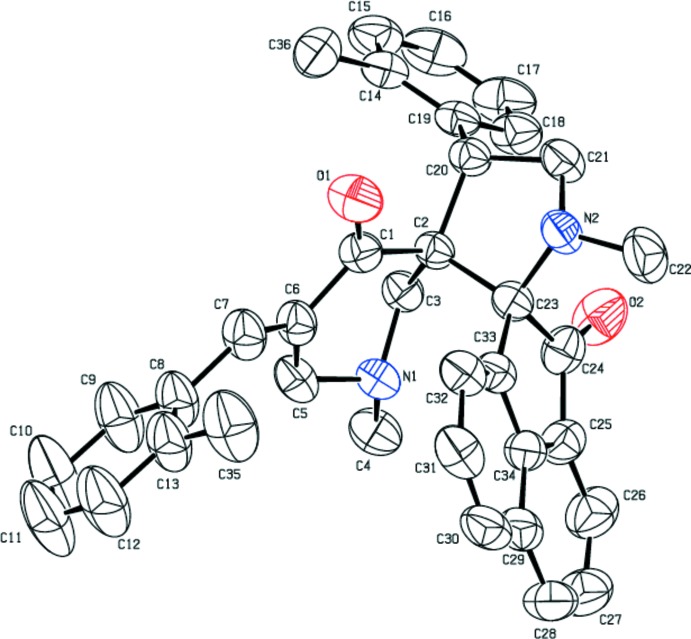
Displacement ellipsoid plot drawn at 50% probability level for (II), showing the atom-labelling scheme. H atoms have been omitted for clarity.

**Figure 7:**
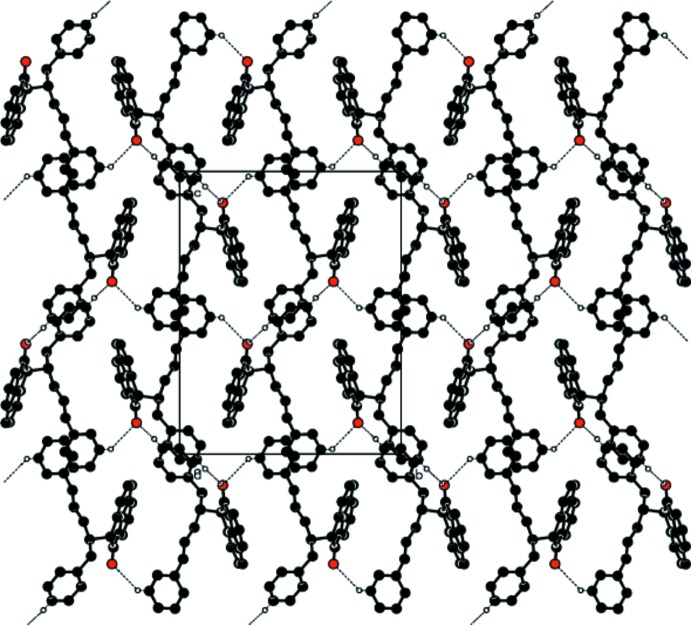
A view of the crystal structure of (II) showing the 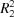
(20) graph-set motifs. Dashed lines indicate weak C—H⋯O inter­molecular inter­actions. H atoms not inter­actions have been omitted for clarity.

**Figure 9:**
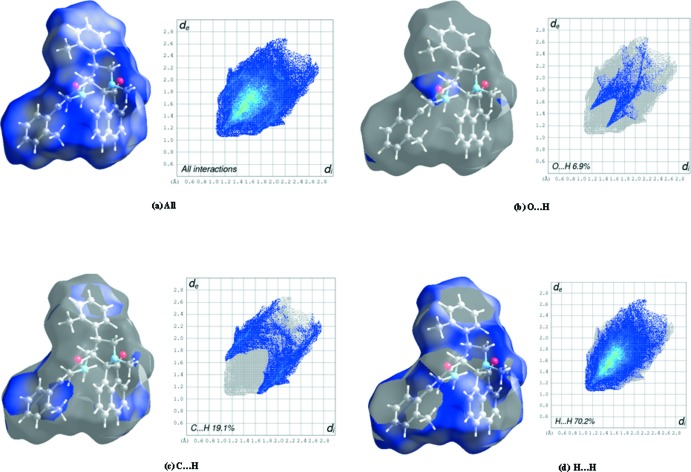
Hirshfeld surface of (II) mapped over shape-index and *d*_norm_ and decomposed fingerprint plots of the dominant inter­actions.

**Table 1:** Selected torsion angles (°) for (I)

O1—C1—C6—C7	−14.3 (2)	Cl2—C14—C19—C20	−0.9 (2)
C7—C8—C13—Cl1	−2.7 (3)	N2—C23—C24—O2	−51.3 (2)

**Table 2:** Selected torsion angles (°) for (II)

O1—C1—C6—C7	−12.8 (3)	C36—C14—C19—C20	−0.1 (3)
C7—C8—C13—C35	−3.6 (4)	N2—C23—C24—O2	−51.5 (2)

**Table 3:** Hydrogen-bond geometry (Å, °) for (I) *Cg*1 is the centroid of the C25–C29/C34 ring.

*D*—H⋯*A*	*D*—H	H⋯*A*	*D*⋯*A*	*D*—H⋯*A*
C10—H10⋯O2^i^	0.93	2.74	3.492 (3)	139
C16—H16⋯O2^ii^	0.93	2.76	3.481 (3)	135
C5—H5*B*⋯*Cg*1^i^	0.97	2.99	3.9466 (19)	168

Symmetry codes: (i) 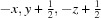; (ii) 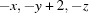.

**Table 4:** Hydrogen-bond geometry (Å, °) for (II) *Cg*2 is the centroid of the C8–C13 ring.

*D*—H⋯*A*	*D*—H	H⋯*A*	*D*⋯*A*	*D*—H⋯*A*
C36—H36*A*⋯O1	0.96	2.66	3.586 (3)	161
C10—H10⋯O2^i^	0.93	2.77	3.529 (3)	140
C16—H16⋯O2^ii^	0.93	2.79	3.530 (3)	137
C35—H35*F*⋯*Cg*2^iii^	0.96	2.94	3.805 (4)	151

Symmetry codes: (i) 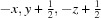; (ii) 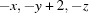; (iii) 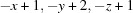.

**Table 5:** Experimental details

	(I)	(II)
Crystal data		
Chemical formula	C_34_H_28_Cl_2_N_2_O_2_	C_36_H_34_N_2_O_2_
*M*_r_	567.48	526.65
Crystal system, space group	Monoclinic, *P*2_1_/*c*	Monoclinic, *P*2_1_/*c*
Temperature (K)	293	293
*a*, *b*, *c* (Å)	8.6710 (4), 15.6756 (7), 20.2284 (9)	8.7507 (5), 15.9089 (8), 20.2879 (10)
β (°)	93.036 (2)	92.935 (2)
*V* (Å^3^)	2745.6 (2)	2820.7 (3)
*Z*	4	4
Radiation type	Mo *K*α	Mo *K*α
μ (mm^−1^)	0.27	0.08
Crystal size (mm)	0.31 × 0.22 × 0.19	0.32 × 0.24 × 0.18

Data collection		
Diffractometer	Bruker SMART APEXII CCD	Bruker SMART APEXII CCD
Absorption correction	Multi-scan (*SADABS*; Bruker, 2001 ▸)	Multi-scan (*SADABS*; Bruker, 2001 ▸)
*T*_min_, *T*_max_	0.771, 1.000	0.816, 1.000
No. of measured, independent and observed [*I* > 2σ(*I*)] reflections	33739, 7274, 5025	28946, 5339, 3349
*R*_int_	0.026	0.040
(sin θ/λ)_max_ (Å^−1^)	0.683	0.610

Refinement		
*R*[*F* ^2^ > 2σ(*F* ^2^)], *wR*(*F* ^2^), *S*	0.044, 0.122, 1.02	0.045, 0.121, 1.02
No. of reflections	7274	5339
No. of parameters	361	362
H-atom treatment	H-atom parameters constrained	H-atom parameters constrained
Δρ_max_, Δρ_min_ (e Å^−3^)	0.33, −0.46	0.17, −0.15

Computer programs: *APEX2* and *SAINT* (Bruker, 2012 ▸), *SHELXS2013* (Sheldrick, 2008 ▸), *SHELXL2018* (Sheldrick, 2015 ▸), *PLATON* (Spek, 2009 ▸) and *publCIF* (Westrip, 2010 ▸).

## supplementary crystallographic information

### 5''-(2-Chlorobenzylidene)-4'-(2-chlorophenyl)-1'-methyldispiro[acenaphthene-1,2'-pyrrolidine-3',3''-piperidine]-2,4''-dione (I) Crystal data

**Table:**

C_34_H_28_Cl_2_N_2_O_2_	*F*(000) = 1184
*M**_r_* = 567.48	*D*_x_ = 1.373 Mg m^−^^3^*D*_m_ = 1.37 Mg m^−^^3^*D*_m_ measured by floatation method
Monoclinic, *P*2_1_/*c*	Mo *K*α radiation, λ = 0.71073 Å
*a* = 8.6710 (4) Å	Cell parameters from 7274 reflections
*b* = 15.6756 (7) Å	θ = 4.8–57.2°
*c* = 20.2284 (9) Å	µ = 0.27 mm^−^^1^
β = 93.036 (2)°	*T* = 293 K
*V* = 2745.6 (2) Å^3^	Block, yellow
*Z* = 4	0.31 × 0.22 × 0.19 mm

### 5''-(2-Chlorobenzylidene)-4'-(2-chlorophenyl)-1'-methyldispiro[acenaphthene-1,2'-pyrrolidine-3',3''-piperidine]-2,4''-dione (I) Data collection

**Table:**

Bruker SMART APEXII CCD diffractometer	5025 reflections with *I* > 2σ(*I*)
Radiation source: fine-focus sealed tube	*R*_int_ = 0.026
φ and ω scans	θ_max_ = 29.1°, θ_min_ = 2.4°
Absorption correction: multi-scan (*SADABS*; Bruker, 2001)	*h* = −11→11
*T*_min_ = 0.771, *T*_max_ = 1.000	*k* = −21→21
33739 measured reflections	*l* = −27→27
7274 independent reflections	

### 5''-(2-Chlorobenzylidene)-4'-(2-chlorophenyl)-1'-methyldispiro[acenaphthene-1,2'-pyrrolidine-3',3''-piperidine]-2,4''-dione (I) Refinement

**Table:**

Refinement on *F*^2^	0 restraints
Least-squares matrix: full	Hydrogen site location: inferred from neighbouring sites
*R*[*F*^2^ > 2σ(*F*^2^)] = 0.044	H-atom parameters constrained
*wR*(*F*^2^) = 0.122	*w* = 1/[σ^2^(*F*_o_^2^) + (0.0458*P*)^2^ + 1.2817*P*] where *P* = (*F*_o_^2^ + 2*F*_c_^2^)/3
*S* = 1.01	(Δ/σ)_max_ = 0.001
7274 reflections	Δρ_max_ = 0.33 e Å^−^^3^
361 parameters	Δρ_min_ = −0.46 e Å^−^^3^

### 5''-(2-Chlorobenzylidene)-4'-(2-chlorophenyl)-1'-methyldispiro[acenaphthene-1,2'-pyrrolidine-3',3''-piperidine]-2,4''-dione (I) Special details

**Table:**

Geometry. All esds (except the esd in the dihedral angle between two l.s. planes) are estimated using the full covariance matrix. The cell esds are taken into account individually in the estimation of esds in distances, angles and torsion angles; correlations between esds in cell parameters are only used when they are defined by crystal symmetry. An approximate (isotropic) treatment of cell esds is used for estimating esds involving l.s. planes.

### 5''-(2-Chlorobenzylidene)-4'-(2-chlorophenyl)-1'-methyldispiro[acenaphthene-1,2'-pyrrolidine-3',3''-piperidine]-2,4''-dione (I) Fractional atomic coordinates and isotropic or equivalent isotropic displacement parameters (Å^2^)

**Table:**

	*x*	*y*	*z*	*U*_iso_*/*U*_eq_	
Cl1	0.38200 (9)	0.88710 (4)	0.49943 (3)	0.0749 (2)	
Cl2	0.53437 (6)	1.07386 (3)	0.14315 (3)	0.05500 (14)	
O1	0.48210 (14)	0.92211 (9)	0.26952 (6)	0.0509 (3)	
O2	0.01681 (16)	0.80480 (10)	0.10859 (7)	0.0587 (4)	
N1	0.02281 (15)	0.96435 (9)	0.23726 (6)	0.0345 (3)	
N2	0.32804 (16)	0.76391 (9)	0.17821 (7)	0.0379 (3)	
C1	0.34543 (18)	0.93585 (10)	0.26001 (8)	0.0339 (3)	
C2	0.25834 (17)	0.90781 (10)	0.19571 (7)	0.0298 (3)	
C3	0.12354 (18)	0.96919 (10)	0.18237 (8)	0.0330 (3)	
H3A	0.161841	1.026946	0.177987	0.040*	
H3B	0.066923	0.953767	0.141473	0.040*	
C4	−0.1310 (2)	0.99694 (13)	0.21990 (10)	0.0486 (4)	
H4A	−0.175090	0.965861	0.182623	0.073*	
H4B	−0.194961	0.990171	0.256864	0.073*	
H4C	−0.124570	1.056321	0.208758	0.073*	
C5	0.0931 (2)	1.00991 (11)	0.29351 (8)	0.0412 (4)	
H5A	0.029809	1.002742	0.331249	0.049*	
H5B	0.096552	1.070296	0.283217	0.049*	
C6	0.2542 (2)	0.97887 (10)	0.31150 (8)	0.0367 (3)	
C7	0.3209 (2)	0.98384 (12)	0.37223 (9)	0.0453 (4)	
H7	0.419755	0.961011	0.377724	0.054*	
C8	0.2571 (2)	1.02102 (12)	0.43141 (9)	0.0498 (5)	
C9	0.1760 (3)	1.09734 (15)	0.42992 (11)	0.0700 (7)	
H9	0.157858	1.125378	0.389746	0.084*	
C10	0.1219 (4)	1.13245 (17)	0.48645 (13)	0.0900 (9)	
H10	0.067945	1.183728	0.484082	0.108*	
C11	0.1467 (4)	1.09265 (18)	0.54595 (12)	0.0925 (10)	
H11	0.109239	1.116503	0.584021	0.111*	
C12	0.2269 (3)	1.01755 (16)	0.54950 (11)	0.0761 (7)	
H12	0.244309	0.990238	0.590018	0.091*	
C13	0.2817 (3)	0.98246 (13)	0.49292 (9)	0.0546 (5)	
C14	0.40575 (19)	1.04221 (11)	0.07931 (8)	0.0395 (4)	
C15	0.3746 (2)	1.09964 (13)	0.02837 (10)	0.0524 (5)	
H15	0.424605	1.152112	0.028284	0.063*	
C16	0.2695 (3)	1.07856 (15)	−0.02190 (10)	0.0604 (6)	
H16	0.247308	1.116892	−0.056185	0.073*	
C17	0.1973 (2)	1.00115 (16)	−0.02157 (10)	0.0589 (6)	
H17	0.125775	0.986900	−0.055648	0.071*	
C18	0.2301 (2)	0.94401 (13)	0.02908 (9)	0.0475 (4)	
H18	0.180273	0.891469	0.028376	0.057*	
C19	0.33571 (18)	0.96284 (11)	0.08114 (8)	0.0355 (3)	
C20	0.37056 (18)	0.90127 (10)	0.13774 (7)	0.0333 (3)	
H20	0.474518	0.914578	0.156241	0.040*	
C21	0.3698 (2)	0.80722 (11)	0.11831 (9)	0.0418 (4)	
H21A	0.294485	0.796255	0.082138	0.050*	
H21B	0.470866	0.789228	0.105323	0.050*	
C22	0.3020 (3)	0.67327 (12)	0.17056 (11)	0.0576 (5)	
H22A	0.275049	0.649399	0.212106	0.086*	
H22B	0.219323	0.663728	0.137946	0.086*	
H22C	0.394350	0.646490	0.156564	0.086*	
C23	0.20239 (17)	0.81269 (10)	0.20545 (8)	0.0322 (3)	
C24	0.0411 (2)	0.79856 (11)	0.16774 (9)	0.0410 (4)	
C25	−0.06990 (19)	0.77207 (11)	0.21615 (10)	0.0429 (4)	
C26	−0.2263 (2)	0.75748 (13)	0.21054 (12)	0.0572 (5)	
H26	−0.281931	0.764906	0.170370	0.069*	
C27	−0.2990 (2)	0.73129 (15)	0.26675 (14)	0.0684 (7)	
H27	−0.405325	0.722892	0.264134	0.082*	
C28	−0.2189 (3)	0.71761 (13)	0.32545 (13)	0.0628 (6)	
H28	−0.271567	0.698729	0.361561	0.075*	
C29	−0.0578 (2)	0.73134 (11)	0.33300 (10)	0.0461 (4)	
C30	0.0409 (3)	0.71831 (12)	0.38919 (10)	0.0526 (5)	
H30	0.001188	0.698030	0.428006	0.063*	
C31	0.1947 (2)	0.73521 (12)	0.38725 (9)	0.0496 (5)	
H31	0.258210	0.724857	0.424875	0.060*	
C32	0.2616 (2)	0.76802 (11)	0.32996 (9)	0.0410 (4)	
H32	0.366809	0.779746	0.330281	0.049*	
C33	0.16946 (18)	0.78193 (10)	0.27480 (8)	0.0339 (3)	
C34	0.01184 (19)	0.76132 (10)	0.27645 (9)	0.0379 (4)	

### 5''-(2-Chlorobenzylidene)-4'-(2-chlorophenyl)-1'-methyldispiro[acenaphthene-1,2'-pyrrolidine-3',3''-piperidine]-2,4''-dione (I) Atomic displacement parameters (Å^2^)

**Table:**

	*U*^11^	*U*^22^	*U*^33^	*U*^12^	*U*^13^	*U*^23^
Cl1	0.1164 (5)	0.0651 (4)	0.0426 (3)	0.0162 (3)	−0.0028 (3)	0.0052 (2)
Cl2	0.0520 (3)	0.0486 (3)	0.0634 (3)	−0.0065 (2)	−0.0062 (2)	−0.0027 (2)
O1	0.0375 (6)	0.0762 (9)	0.0382 (7)	0.0023 (6)	−0.0059 (5)	−0.0024 (6)
O2	0.0606 (8)	0.0712 (10)	0.0422 (8)	−0.0164 (7)	−0.0156 (6)	0.0065 (7)
N1	0.0344 (7)	0.0376 (7)	0.0316 (7)	0.0049 (5)	0.0030 (5)	−0.0007 (6)
N2	0.0460 (8)	0.0329 (7)	0.0352 (8)	0.0044 (6)	0.0067 (6)	−0.0027 (6)
C1	0.0384 (8)	0.0354 (8)	0.0275 (8)	−0.0033 (6)	−0.0013 (6)	0.0035 (6)
C2	0.0309 (7)	0.0335 (8)	0.0252 (7)	0.0007 (6)	0.0013 (6)	0.0008 (6)
C3	0.0365 (8)	0.0355 (8)	0.0268 (8)	0.0039 (6)	−0.0005 (6)	0.0016 (6)
C4	0.0411 (9)	0.0537 (11)	0.0511 (11)	0.0131 (8)	0.0035 (8)	0.0005 (9)
C5	0.0517 (10)	0.0408 (9)	0.0315 (9)	0.0077 (7)	0.0039 (7)	−0.0038 (7)
C6	0.0465 (9)	0.0344 (8)	0.0291 (8)	−0.0030 (7)	0.0010 (7)	−0.0008 (6)
C7	0.0571 (11)	0.0466 (10)	0.0318 (9)	−0.0019 (8)	−0.0023 (8)	−0.0012 (7)
C8	0.0732 (13)	0.0468 (10)	0.0288 (9)	−0.0039 (9)	−0.0025 (8)	−0.0063 (8)
C9	0.117 (2)	0.0542 (13)	0.0381 (11)	0.0107 (13)	−0.0047 (12)	−0.0076 (9)
C10	0.153 (3)	0.0611 (15)	0.0555 (15)	0.0301 (16)	0.0057 (16)	−0.0159 (12)
C11	0.163 (3)	0.0710 (17)	0.0448 (14)	0.0170 (18)	0.0209 (16)	−0.0168 (12)
C12	0.133 (2)	0.0629 (14)	0.0337 (11)	0.0001 (15)	0.0128 (13)	−0.0019 (10)
C13	0.0821 (14)	0.0460 (11)	0.0356 (10)	−0.0045 (10)	0.0017 (9)	−0.0034 (8)
C14	0.0358 (8)	0.0465 (9)	0.0369 (9)	0.0042 (7)	0.0078 (7)	0.0023 (7)
C15	0.0570 (11)	0.0509 (11)	0.0507 (12)	0.0045 (9)	0.0165 (9)	0.0135 (9)
C16	0.0635 (13)	0.0777 (15)	0.0409 (11)	0.0173 (11)	0.0104 (9)	0.0262 (10)
C17	0.0525 (11)	0.0899 (17)	0.0337 (10)	0.0036 (11)	−0.0023 (8)	0.0126 (10)
C18	0.0463 (10)	0.0642 (12)	0.0319 (9)	−0.0031 (8)	0.0010 (7)	0.0051 (8)
C19	0.0337 (8)	0.0459 (9)	0.0275 (8)	0.0032 (7)	0.0065 (6)	0.0027 (7)
C20	0.0324 (7)	0.0403 (8)	0.0273 (8)	0.0011 (6)	0.0013 (6)	0.0002 (6)
C21	0.0485 (9)	0.0432 (9)	0.0343 (9)	0.0054 (7)	0.0075 (7)	−0.0040 (7)
C22	0.0804 (14)	0.0358 (10)	0.0577 (13)	0.0030 (9)	0.0133 (11)	−0.0059 (9)
C23	0.0335 (7)	0.0336 (8)	0.0292 (8)	0.0001 (6)	−0.0006 (6)	0.0014 (6)
C24	0.0413 (9)	0.0364 (9)	0.0443 (10)	−0.0033 (7)	−0.0059 (7)	0.0012 (7)
C25	0.0367 (8)	0.0342 (9)	0.0574 (11)	−0.0009 (7)	−0.0002 (8)	0.0006 (8)
C26	0.0391 (9)	0.0519 (11)	0.0798 (15)	−0.0016 (8)	−0.0034 (10)	−0.0004 (10)
C27	0.0395 (10)	0.0635 (14)	0.104 (2)	−0.0058 (9)	0.0173 (12)	−0.0037 (13)
C28	0.0549 (12)	0.0518 (12)	0.0851 (17)	−0.0068 (9)	0.0344 (12)	−0.0017 (11)
C29	0.0539 (10)	0.0309 (8)	0.0555 (12)	0.0016 (7)	0.0216 (9)	0.0003 (8)
C30	0.0744 (13)	0.0377 (9)	0.0482 (11)	0.0050 (9)	0.0253 (10)	0.0054 (8)
C31	0.0711 (13)	0.0406 (10)	0.0374 (10)	0.0110 (9)	0.0051 (9)	0.0074 (8)
C32	0.0461 (9)	0.0382 (9)	0.0389 (9)	0.0067 (7)	0.0033 (7)	0.0048 (7)
C33	0.0377 (8)	0.0288 (7)	0.0355 (9)	0.0029 (6)	0.0049 (6)	0.0026 (6)
C34	0.0403 (8)	0.0261 (7)	0.0479 (10)	0.0012 (6)	0.0090 (7)	0.0000 (7)

### 5''-(2-Chlorobenzylidene)-4'-(2-chlorophenyl)-1'-methyldispiro[acenaphthene-1,2'-pyrrolidine-3',3''-piperidine]-2,4''-dione (I) Geometric parameters (Å, º)

**Table:**

Cl1—C13	1.731 (2)	C14—C19	1.386 (2)
Cl2—C14	1.7336 (18)	C15—C16	1.369 (3)
O1—C1	1.2098 (19)	C15—H15	0.9300
O2—C24	1.208 (2)	C16—C17	1.365 (3)
N1—C5	1.450 (2)	C16—H16	0.9300
N1—C3	1.4506 (19)	C17—C18	1.379 (3)
N1—C4	1.454 (2)	C17—H17	0.9300
N2—C22	1.446 (2)	C18—C19	1.390 (2)
N2—C21	1.451 (2)	C18—H18	0.9300
N2—C23	1.462 (2)	C19—C20	1.516 (2)
C1—C6	1.501 (2)	C20—C21	1.526 (2)
C1—C2	1.533 (2)	C20—H20	0.9800
C2—C3	1.527 (2)	C21—H21A	0.9700
C2—C20	1.566 (2)	C21—H21B	0.9700
C2—C23	1.584 (2)	C22—H22A	0.9600
C3—H3A	0.9700	C22—H22B	0.9600
C3—H3B	0.9700	C22—H22C	0.9600
C4—H4A	0.9600	C23—C33	1.525 (2)
C4—H4B	0.9600	C23—C24	1.573 (2)
C4—H4C	0.9600	C24—C25	1.469 (3)
C5—C6	1.505 (2)	C25—C26	1.374 (2)
C5—H5A	0.9700	C25—C34	1.388 (3)
C5—H5B	0.9700	C26—C27	1.391 (3)
C6—C7	1.332 (2)	C26—H26	0.9300
C7—C8	1.466 (3)	C27—C28	1.360 (3)
C7—H7	0.9300	C27—H27	0.9300
C8—C9	1.387 (3)	C28—C29	1.414 (3)
C8—C13	1.390 (3)	C28—H28	0.9300
C9—C10	1.374 (3)	C29—C30	1.401 (3)
C9—H9	0.9300	C29—C34	1.402 (2)
C10—C11	1.363 (4)	C30—C31	1.362 (3)
C10—H10	0.9300	C30—H30	0.9300
C11—C12	1.367 (4)	C31—C32	1.420 (2)
C11—H11	0.9300	C31—H31	0.9300
C12—C13	1.377 (3)	C32—C33	1.355 (2)
C12—H12	0.9300	C32—H32	0.9300
C14—C15	1.384 (3)	C33—C34	1.406 (2)
			
C5—N1—C3	109.25 (13)	C16—C17—C18	120.3 (2)
C5—N1—C4	111.01 (13)	C16—C17—H17	119.9
C3—N1—C4	112.25 (13)	C18—C17—H17	119.9
C22—N2—C21	114.51 (14)	C17—C18—C19	121.78 (19)
C22—N2—C23	116.03 (14)	C17—C18—H18	119.1
C21—N2—C23	107.08 (13)	C19—C18—H18	119.1
O1—C1—C6	121.17 (15)	C14—C19—C18	116.24 (16)
O1—C1—C2	121.21 (15)	C14—C19—C20	121.46 (15)
C6—C1—C2	117.60 (13)	C18—C19—C20	122.29 (16)
C3—C2—C1	107.89 (12)	C19—C20—C21	115.01 (13)
C3—C2—C20	114.15 (12)	C19—C20—C2	114.64 (12)
C1—C2—C20	110.87 (12)	C21—C20—C2	105.17 (13)
C3—C2—C23	112.25 (12)	C19—C20—H20	107.2
C1—C2—C23	107.74 (12)	C21—C20—H20	107.2
C20—C2—C23	103.77 (12)	C2—C20—H20	107.2
N1—C3—C2	108.55 (12)	N2—C21—C20	103.58 (13)
N1—C3—H3A	110.0	N2—C21—H21A	111.0
C2—C3—H3A	110.0	C20—C21—H21A	111.0
N1—C3—H3B	110.0	N2—C21—H21B	111.0
C2—C3—H3B	110.0	C20—C21—H21B	111.0
H3A—C3—H3B	108.4	H21A—C21—H21B	109.0
N1—C4—H4A	109.5	N2—C22—H22A	109.5
N1—C4—H4B	109.5	N2—C22—H22B	109.5
H4A—C4—H4B	109.5	H22A—C22—H22B	109.5
N1—C4—H4C	109.5	N2—C22—H22C	109.5
H4A—C4—H4C	109.5	H22A—C22—H22C	109.5
H4B—C4—H4C	109.5	H22B—C22—H22C	109.5
N1—C5—C6	112.12 (13)	N2—C23—C33	111.16 (12)
N1—C5—H5A	109.2	N2—C23—C24	113.88 (13)
C6—C5—H5A	109.2	C33—C23—C24	101.34 (12)
N1—C5—H5B	109.2	N2—C23—C2	101.90 (12)
C6—C5—H5B	109.2	C33—C23—C2	119.06 (13)
H5A—C5—H5B	107.9	C24—C23—C2	110.04 (12)
C7—C6—C1	116.54 (16)	O2—C24—C25	126.70 (16)
C7—C6—C5	123.80 (16)	O2—C24—C23	124.93 (16)
C1—C6—C5	119.60 (14)	C25—C24—C23	108.28 (14)
C6—C7—C8	127.74 (18)	C26—C25—C34	120.53 (18)
C6—C7—H7	116.1	C26—C25—C24	132.12 (19)
C8—C7—H7	116.1	C34—C25—C24	107.35 (15)
C9—C8—C13	116.62 (18)	C25—C26—C27	118.0 (2)
C9—C8—C7	122.54 (18)	C25—C26—H26	121.0
C13—C8—C7	120.78 (18)	C27—C26—H26	121.0
C10—C9—C8	121.5 (2)	C28—C27—C26	121.8 (2)
C10—C9—H9	119.2	C28—C27—H27	119.1
C8—C9—H9	119.2	C26—C27—H27	119.1
C11—C10—C9	120.5 (2)	C27—C28—C29	121.7 (2)
C11—C10—H10	119.8	C27—C28—H28	119.1
C9—C10—H10	119.8	C29—C28—H28	119.1
C10—C11—C12	119.8 (2)	C30—C29—C34	116.10 (17)
C10—C11—H11	120.1	C30—C29—C28	128.53 (19)
C12—C11—H11	120.1	C34—C29—C28	115.4 (2)
C11—C12—C13	119.8 (2)	C31—C30—C29	120.33 (17)
C11—C12—H12	120.1	C31—C30—H30	119.8
C13—C12—H12	120.1	C29—C30—H30	119.8
C12—C13—C8	121.8 (2)	C30—C31—C32	122.50 (18)
C12—C13—Cl1	118.43 (17)	C30—C31—H31	118.8
C8—C13—Cl1	119.76 (15)	C32—C31—H31	118.8
C15—C14—C19	122.37 (17)	C33—C32—C31	118.77 (17)
C15—C14—Cl2	117.45 (15)	C33—C32—H32	120.6
C19—C14—Cl2	120.17 (13)	C31—C32—H32	120.6
C16—C15—C14	119.5 (2)	C32—C33—C34	118.36 (15)
C16—C15—H15	120.3	C32—C33—C23	132.64 (15)
C14—C15—H15	120.3	C34—C33—C23	108.90 (14)
C17—C16—C15	119.85 (19)	C25—C34—C29	122.39 (17)
C17—C16—H16	120.1	C25—C34—C33	113.76 (15)
C15—C16—H16	120.1	C29—C34—C33	123.85 (17)
			
O1—C1—C2—C3	−151.50 (15)	C19—C20—C21—N2	−152.40 (13)
C6—C1—C2—C3	30.10 (18)	C2—C20—C21—N2	−25.30 (16)
O1—C1—C2—C20	−25.8 (2)	C22—N2—C23—C33	60.97 (19)
C6—C1—C2—C20	155.77 (13)	C21—N2—C23—C33	−169.74 (13)
O1—C1—C2—C23	87.09 (18)	C22—N2—C23—C24	−52.7 (2)
C6—C1—C2—C23	−91.31 (15)	C21—N2—C23—C24	76.57 (16)
C5—N1—C3—C2	75.82 (16)	C22—N2—C23—C2	−171.16 (15)
C4—N1—C3—C2	−160.58 (14)	C21—N2—C23—C2	−41.87 (15)
C1—C2—C3—N1	−60.62 (16)	C3—C2—C23—N2	147.45 (12)
C20—C2—C3—N1	175.67 (13)	C1—C2—C23—N2	−93.90 (14)
C23—C2—C3—N1	57.94 (16)	C20—C2—C23—N2	23.71 (14)
C3—N1—C5—C6	−54.27 (18)	C3—C2—C23—C33	−89.93 (16)
C4—N1—C5—C6	−178.59 (14)	C1—C2—C23—C33	28.72 (17)
O1—C1—C6—C7	−14.3 (2)	C20—C2—C23—C33	146.34 (13)
C2—C1—C6—C7	164.07 (15)	C3—C2—C23—C24	26.31 (17)
O1—C1—C6—C5	168.41 (16)	C1—C2—C23—C24	144.96 (13)
C2—C1—C6—C5	−13.2 (2)	C20—C2—C23—C24	−97.43 (14)
N1—C5—C6—C7	−153.17 (16)	N2—C23—C24—O2	−51.3 (2)
N1—C5—C6—C1	23.9 (2)	C33—C23—C24—O2	−170.73 (17)
C1—C6—C7—C8	−179.00 (17)	C2—C23—C24—O2	62.4 (2)
C5—C6—C7—C8	−1.9 (3)	N2—C23—C24—C25	125.47 (15)
C6—C7—C8—C9	−42.2 (3)	C33—C23—C24—C25	6.04 (16)
C6—C7—C8—C13	140.7 (2)	C2—C23—C24—C25	−120.86 (14)
C13—C8—C9—C10	−0.5 (4)	O2—C24—C25—C26	−8.4 (3)
C7—C8—C9—C10	−177.7 (3)	C23—C24—C25—C26	174.85 (19)
C8—C9—C10—C11	−0.1 (5)	O2—C24—C25—C34	171.10 (18)
C9—C10—C11—C12	0.4 (5)	C23—C24—C25—C34	−5.60 (18)
C10—C11—C12—C13	−0.2 (5)	C34—C25—C26—C27	−0.4 (3)
C11—C12—C13—C8	−0.4 (4)	C24—C25—C26—C27	179.1 (2)
C11—C12—C13—Cl1	−179.7 (2)	C25—C26—C27—C28	−2.0 (3)
C9—C8—C13—C12	0.8 (3)	C26—C27—C28—C29	1.6 (3)
C7—C8—C13—C12	178.0 (2)	C27—C28—C29—C30	−178.3 (2)
C9—C8—C13—Cl1	−179.95 (18)	C27—C28—C29—C34	1.2 (3)
C7—C8—C13—Cl1	−2.7 (3)	C34—C29—C30—C31	0.5 (3)
C19—C14—C15—C16	0.9 (3)	C28—C29—C30—C31	179.99 (19)
Cl2—C14—C15—C16	−177.66 (15)	C29—C30—C31—C32	1.4 (3)
C14—C15—C16—C17	−0.4 (3)	C30—C31—C32—C33	−1.0 (3)
C15—C16—C17—C18	−0.2 (3)	C31—C32—C33—C34	−1.4 (2)
C16—C17—C18—C19	0.3 (3)	C31—C32—C33—C23	−177.36 (16)
C15—C14—C19—C18	−0.7 (2)	N2—C23—C33—C32	50.4 (2)
Cl2—C14—C19—C18	177.79 (13)	C24—C23—C33—C32	171.78 (17)
C15—C14—C19—C20	−179.46 (15)	C2—C23—C33—C32	−67.5 (2)
Cl2—C14—C19—C20	−0.9 (2)	N2—C23—C33—C34	−125.80 (14)
C17—C18—C19—C14	0.1 (3)	C24—C23—C33—C34	−4.45 (16)
C17—C18—C19—C20	178.84 (17)	C2—C23—C33—C34	116.30 (15)
C14—C19—C20—C21	−145.35 (15)	C26—C25—C34—C29	3.3 (3)
C18—C19—C20—C21	36.0 (2)	C24—C25—C34—C29	−176.29 (15)
C14—C19—C20—C2	92.53 (18)	C26—C25—C34—C33	−177.61 (16)
C18—C19—C20—C2	−86.13 (19)	C24—C25—C34—C33	2.8 (2)
C3—C2—C20—C19	5.57 (19)	C30—C29—C34—C25	175.93 (16)
C1—C2—C20—C19	−116.52 (15)	C28—C29—C34—C25	−3.6 (2)
C23—C2—C20—C19	128.06 (14)	C30—C29—C34—C33	−3.0 (2)
C3—C2—C20—C21	−121.76 (14)	C28—C29—C34—C33	177.43 (16)
C1—C2—C20—C21	116.15 (14)	C32—C33—C34—C25	−175.52 (15)
C23—C2—C20—C21	0.73 (15)	C23—C33—C34—C25	1.33 (19)
C22—N2—C21—C20	173.28 (15)	C32—C33—C34—C29	3.5 (2)
C23—N2—C21—C20	43.13 (17)	C23—C33—C34—C29	−179.62 (15)

### 5''-(2-Chlorobenzylidene)-4'-(2-chlorophenyl)-1'-methyldispiro[acenaphthene-1,2'-pyrrolidine-3',3''-piperidine]-2,4''-dione (I) Hydrogen-bond geometry (Å, º)

*Cg*1 is the centroid of the C25–C29/C34 ring.

**Table:**

*D*—H···*A*	*D*—H	H···*A*	*D*···*A*	*D*—H···*A*
C10—H10···O2^i^	0.93	2.74	3.492 (3)	139
C16—H16···O2^ii^	0.93	2.76	3.481 (3)	135
C5—H5*B*···*Cg*1^i^	0.97	2.99	3.9466 (19)	168

Symmetry codes: (i) −*x*, *y*+1/2, −*z*+1/2; (ii) −*x*, −*y*+2, −*z*.

### 1'-Methyl-5''-(2-methylbenzylidene)-4'-(2-methylphenyl)dispiro[acenaphthene-1,2'-pyrrolidine-3',3''-piperidine]-2,4''-dione (II) Crystal data

**Table:**

C_36_H_34_N_2_O_2_	*F*(000) = 1120
*M**_r_* = 526.65	*D*_x_ = 1.240 Mg m^−^^3^*D*_m_ = 1.24 Mg m^−^^3^*D*_m_ measured by floatation method
Monoclinic, *P*2_1_/*c*	Mo *K*α radiation, λ = 0.71073 Å
*a* = 8.7507 (5) Å	Cell parameters from 5358 reflections
*b* = 15.9089 (8) Å	θ = 4.7–42.0°
*c* = 20.2879 (10) Å	µ = 0.08 mm^−^^1^
β = 92.935 (2)°	*T* = 293 K
*V* = 2820.7 (3) Å^3^	Block, yellow
*Z* = 4	0.32 × 0.24 × 0.18 mm

### 1'-Methyl-5''-(2-methylbenzylidene)-4'-(2-methylphenyl)dispiro[acenaphthene-1,2'-pyrrolidine-3',3''-piperidine]-2,4''-dione (II) Data collection

**Table:**

Bruker SMART APEXII CCD diffractometer	3349 reflections with *I* > 2σ(*I*)
Radiation source: fine-focus sealed tube	*R*_int_ = 0.040
φ and ω scans	θ_max_ = 25.7°, θ_min_ = 2.3°
Absorption correction: multi-scan (*SADABS*; Bruker, 2001)	*h* = −10→10
*T*_min_ = 0.816, *T*_max_ = 1.000	*k* = −19→19
28946 measured reflections	*l* = −24→18
5339 independent reflections	

### 1'-Methyl-5''-(2-methylbenzylidene)-4'-(2-methylphenyl)dispiro[acenaphthene-1,2'-pyrrolidine-3',3''-piperidine]-2,4''-dione (II) Refinement

**Table:**

Refinement on *F*^2^	Hydrogen site location: inferred from neighbouring sites
Least-squares matrix: full	H-atom parameters constrained
*R*[*F*^2^ > 2σ(*F*^2^)] = 0.045	*w* = 1/[σ^2^(*F*_o_^2^) + (0.0506*P*)^2^ + 0.5169*P*] where *P* = (*F*_o_^2^ + 2*F*_c_^2^)/3
*wR*(*F*^2^) = 0.121	(Δ/σ)_max_ = 0.001
*S* = 1.02	Δρ_max_ = 0.17 e Å^−^^3^
5339 reflections	Δρ_min_ = −0.15 e Å^−^^3^
362 parameters	Extinction correction: SHELXL2018 (Sheldrick, 2015), Fc^*^=kFc[1+0.001xFc^2^λ^3^/sin(2θ)]^-1/4^
0 restraints	Extinction coefficient: 0.0040 (6)

### 1'-Methyl-5''-(2-methylbenzylidene)-4'-(2-methylphenyl)dispiro[acenaphthene-1,2'-pyrrolidine-3',3''-piperidine]-2,4''-dione (II) Special details

**Table:**

Geometry. All esds (except the esd in the dihedral angle between two l.s. planes) are estimated using the full covariance matrix. The cell esds are taken into account individually in the estimation of esds in distances, angles and torsion angles; correlations between esds in cell parameters are only used when they are defined by crystal symmetry. An approximate (isotropic) treatment of cell esds is used for estimating esds involving l.s. planes.

### 1'-Methyl-5''-(2-methylbenzylidene)-4'-(2-methylphenyl)dispiro[acenaphthene-1,2'-pyrrolidine-3',3''-piperidine]-2,4''-dione (II) Fractional atomic coordinates and isotropic or equivalent isotropic displacement parameters (Å^2^)

**Table:**

	*x*	*y*	*z*	*U*_iso_*/*U*_eq_	Occ. (<1)
O1	0.47706 (15)	0.91884 (10)	0.26949 (6)	0.0632 (4)	
O2	0.01075 (17)	0.80552 (10)	0.11060 (7)	0.0749 (5)	
N1	0.02197 (15)	0.96279 (9)	0.23966 (7)	0.0438 (4)	
N2	0.32038 (17)	0.76401 (9)	0.17891 (7)	0.0488 (4)	
C1	0.3410 (2)	0.93366 (11)	0.26053 (8)	0.0434 (4)	
C2	0.25341 (18)	0.90633 (11)	0.19667 (8)	0.0377 (4)	
C3	0.12046 (19)	0.96732 (11)	0.18425 (8)	0.0420 (4)	
H3A	0.159005	1.024095	0.179771	0.050*	
H3B	0.063116	0.952451	0.143748	0.050*	
C4	−0.1307 (2)	0.99492 (14)	0.22319 (10)	0.0619 (6)	
H4A	−0.175538	0.964127	0.186372	0.093*	
H4B	−0.192980	0.988480	0.260484	0.093*	
H4C	−0.124579	1.053358	0.211850	0.093*	
C5	0.0937 (2)	1.00778 (12)	0.29519 (9)	0.0529 (5)	
H5A	0.032387	1.000780	0.333317	0.063*	
H5B	0.096585	1.067257	0.284807	0.063*	
C6	0.2541 (2)	0.97734 (11)	0.31209 (8)	0.0459 (4)	
C7	0.3224 (2)	0.98436 (13)	0.37200 (9)	0.0566 (5)	
H7	0.420638	0.962182	0.376842	0.068*	
C8	0.2630 (3)	1.02252 (14)	0.43153 (9)	0.0637 (6)	
C9	0.1907 (3)	1.09994 (16)	0.42926 (11)	0.0862 (8)	
H9	0.176292	1.127477	0.388985	0.103*	
C10	0.1399 (4)	1.13691 (19)	0.48527 (13)	0.1125 (11)	
H10	0.092416	1.189186	0.482807	0.135*	
C11	0.1595 (4)	1.0966 (2)	0.54474 (13)	0.1158 (11)	
H11	0.124439	1.121064	0.582782	0.139*	
C12	0.2304 (4)	1.02073 (18)	0.54774 (12)	0.1011 (10)	
H12	0.242808	0.993784	0.588346	0.121*	
C13	0.2852 (3)	0.98180 (14)	0.49223 (10)	0.0736 (7)	
C14	0.4072 (2)	1.03916 (12)	0.08230 (9)	0.0477 (5)	
C15	0.3741 (2)	1.09458 (14)	0.03059 (10)	0.0627 (6)	
H15	0.425474	1.145727	0.029917	0.075*	
C16	0.2684 (3)	1.07634 (17)	−0.01936 (11)	0.0716 (7)	
H16	0.247654	1.114850	−0.053129	0.086*	
C17	0.1933 (2)	1.00083 (17)	−0.01915 (10)	0.0693 (6)	
H17	0.121043	0.987782	−0.052809	0.083*	
C18	0.2252 (2)	0.94425 (14)	0.03112 (9)	0.0563 (5)	
H18	0.173963	0.893017	0.030703	0.068*	
C19	0.33151 (19)	0.96161 (12)	0.08224 (8)	0.0429 (4)	
C20	0.36354 (19)	0.89945 (11)	0.13800 (8)	0.0420 (4)	
H20	0.466999	0.911443	0.156377	0.050*	
C21	0.3607 (2)	0.80656 (12)	0.11868 (9)	0.0529 (5)	
H21A	0.284808	0.796067	0.083082	0.063*	
H21B	0.460023	0.788322	0.105068	0.063*	
C22	0.2938 (3)	0.67430 (13)	0.17162 (11)	0.0731 (7)	
H22A	0.268011	0.651006	0.213260	0.110*	
H22B	0.211144	0.664885	0.139531	0.110*	
H22C	0.384812	0.647695	0.157250	0.110*	
C23	0.19669 (19)	0.81255 (11)	0.20669 (8)	0.0422 (4)	
C24	0.0359 (2)	0.79910 (12)	0.16969 (10)	0.0523 (5)	
C25	−0.0735 (2)	0.77254 (12)	0.21815 (10)	0.0540 (5)	
C26	−0.2285 (2)	0.75824 (14)	0.21310 (13)	0.0723 (6)	
H26	−0.284279	0.766562	0.173375	0.087*	
C27	−0.2998 (3)	0.73101 (16)	0.26895 (16)	0.0843 (8)	
H27	−0.405192	0.722827	0.266586	0.101*	
C28	−0.2194 (3)	0.71598 (14)	0.32708 (14)	0.0765 (7)	
H28	−0.270670	0.696290	0.363029	0.092*	
C29	−0.0593 (2)	0.72969 (12)	0.33393 (11)	0.0580 (5)	
C30	0.0395 (3)	0.71575 (13)	0.38964 (11)	0.0661 (6)	
H30	0.000748	0.694837	0.428213	0.079*	
C31	0.1921 (3)	0.73275 (12)	0.38742 (10)	0.0618 (6)	
H31	0.255708	0.721647	0.424532	0.074*	
C32	0.2575 (2)	0.76681 (11)	0.33047 (9)	0.0504 (5)	
H32	0.361543	0.778819	0.330644	0.060*	
C33	0.1650 (2)	0.78149 (11)	0.27577 (8)	0.0430 (4)	
C34	0.0085 (2)	0.76075 (11)	0.27794 (9)	0.0473 (5)	
C35	0.3604 (4)	0.89722 (16)	0.49768 (11)	0.0951 (9)	
H35A	0.391046	0.879874	0.454998	0.143*	0.5
H35B	0.448790	0.900568	0.527609	0.143*	0.5
H35C	0.289565	0.857078	0.513916	0.143*	0.5
H35D	0.361888	0.878473	0.542684	0.143*	0.5
H35E	0.304143	0.857779	0.470073	0.143*	0.5
H35F	0.463369	0.901268	0.483766	0.143*	0.5
C36	0.5214 (2)	1.06401 (14)	0.13658 (11)	0.0659 (6)	
H36A	0.531595	1.019372	0.168344	0.099*	0.5
H36B	0.618648	1.074534	0.118366	0.099*	0.5
H36C	0.486814	1.114002	0.157740	0.099*	0.5
H36D	0.559776	1.119233	0.127956	0.099*	0.5
H36E	0.472723	1.064071	0.177934	0.099*	0.5
H36F	0.604557	1.024604	0.138560	0.099*	0.5

### 1'-Methyl-5''-(2-methylbenzylidene)-4'-(2-methylphenyl)dispiro[acenaphthene-1,2'-pyrrolidine-3',3''-piperidine]-2,4''-dione (II) Atomic displacement parameters (Å^2^)

**Table:**

	*U*^11^	*U*^22^	*U*^33^	*U*^12^	*U*^13^	*U*^23^
O1	0.0476 (8)	0.0952 (11)	0.0459 (8)	0.0049 (7)	−0.0050 (6)	−0.0030 (7)
O2	0.0784 (10)	0.0890 (12)	0.0551 (10)	−0.0227 (8)	−0.0182 (8)	0.0099 (8)
N1	0.0438 (8)	0.0493 (9)	0.0386 (9)	0.0075 (7)	0.0052 (7)	0.0005 (7)
N2	0.0621 (10)	0.0420 (9)	0.0434 (9)	0.0056 (7)	0.0121 (7)	−0.0019 (7)
C1	0.0449 (11)	0.0489 (11)	0.0361 (10)	−0.0008 (8)	0.0004 (8)	0.0042 (8)
C2	0.0396 (9)	0.0432 (10)	0.0303 (9)	0.0010 (8)	0.0026 (7)	0.0004 (8)
C3	0.0471 (10)	0.0452 (10)	0.0336 (10)	0.0022 (8)	0.0018 (8)	0.0018 (8)
C4	0.0526 (12)	0.0685 (14)	0.0650 (14)	0.0164 (10)	0.0071 (10)	−0.0003 (11)
C5	0.0666 (13)	0.0533 (12)	0.0391 (11)	0.0101 (10)	0.0072 (9)	−0.0033 (9)
C6	0.0597 (11)	0.0439 (11)	0.0340 (10)	−0.0015 (9)	0.0009 (8)	0.0001 (8)
C7	0.0757 (14)	0.0559 (12)	0.0377 (11)	−0.0012 (10)	−0.0014 (10)	−0.0005 (9)
C8	0.0948 (16)	0.0596 (14)	0.0362 (12)	0.0032 (12)	−0.0021 (10)	−0.0079 (10)
C9	0.143 (2)	0.0701 (16)	0.0441 (14)	0.0211 (16)	−0.0056 (14)	−0.0085 (12)
C10	0.193 (3)	0.0801 (19)	0.0642 (18)	0.040 (2)	0.0039 (19)	−0.0204 (15)
C11	0.202 (3)	0.093 (2)	0.0542 (17)	0.028 (2)	0.0216 (18)	−0.0222 (16)
C12	0.179 (3)	0.0834 (19)	0.0420 (14)	0.013 (2)	0.0156 (16)	−0.0069 (13)
C13	0.118 (2)	0.0631 (15)	0.0393 (12)	0.0007 (14)	0.0031 (12)	−0.0062 (11)
C14	0.0447 (10)	0.0549 (12)	0.0444 (11)	0.0043 (9)	0.0126 (8)	0.0033 (9)
C15	0.0696 (14)	0.0620 (14)	0.0584 (14)	0.0034 (11)	0.0222 (11)	0.0141 (11)
C16	0.0759 (16)	0.0903 (18)	0.0497 (14)	0.0212 (14)	0.0140 (12)	0.0276 (12)
C17	0.0593 (13)	0.107 (2)	0.0415 (12)	0.0025 (13)	0.0008 (10)	0.0154 (12)
C18	0.0545 (12)	0.0767 (14)	0.0377 (11)	−0.0056 (10)	0.0028 (9)	0.0068 (10)
C19	0.0414 (10)	0.0557 (12)	0.0322 (10)	0.0019 (9)	0.0084 (8)	0.0030 (8)
C20	0.0402 (10)	0.0518 (11)	0.0342 (10)	−0.0004 (8)	0.0025 (7)	−0.0005 (8)
C21	0.0609 (12)	0.0562 (12)	0.0425 (11)	0.0046 (9)	0.0117 (9)	−0.0047 (9)
C22	0.1067 (18)	0.0462 (12)	0.0682 (15)	0.0039 (12)	0.0207 (13)	−0.0071 (11)
C23	0.0437 (10)	0.0457 (11)	0.0372 (10)	0.0020 (8)	0.0029 (8)	0.0007 (8)
C24	0.0573 (12)	0.0463 (11)	0.0524 (13)	−0.0040 (9)	−0.0057 (10)	0.0040 (9)
C25	0.0477 (11)	0.0459 (11)	0.0684 (14)	−0.0011 (9)	0.0045 (10)	0.0018 (10)
C26	0.0524 (13)	0.0648 (14)	0.0994 (19)	−0.0021 (11)	0.0012 (12)	−0.0008 (13)
C27	0.0542 (14)	0.0776 (17)	0.123 (2)	−0.0061 (13)	0.0221 (16)	−0.0055 (17)
C28	0.0690 (16)	0.0648 (15)	0.100 (2)	−0.0065 (12)	0.0418 (15)	−0.0021 (14)
C29	0.0696 (14)	0.0404 (11)	0.0664 (14)	0.0048 (10)	0.0249 (12)	0.0005 (10)
C30	0.0901 (17)	0.0501 (13)	0.0613 (14)	0.0083 (12)	0.0364 (13)	0.0071 (10)
C31	0.0919 (17)	0.0501 (12)	0.0439 (12)	0.0184 (12)	0.0088 (11)	0.0087 (9)
C32	0.0591 (12)	0.0465 (11)	0.0460 (11)	0.0112 (9)	0.0069 (9)	0.0061 (9)
C33	0.0509 (11)	0.0366 (10)	0.0422 (11)	0.0069 (8)	0.0082 (8)	0.0033 (8)
C34	0.0519 (11)	0.0349 (10)	0.0563 (12)	0.0028 (8)	0.0149 (10)	0.0026 (9)
C35	0.157 (3)	0.0752 (17)	0.0521 (14)	0.0178 (17)	−0.0015 (15)	0.0049 (12)
C36	0.0637 (13)	0.0622 (14)	0.0717 (15)	−0.0101 (11)	0.0025 (11)	−0.0023 (11)

### 1'-Methyl-5''-(2-methylbenzylidene)-4'-(2-methylphenyl)dispiro[acenaphthene-1,2'-pyrrolidine-3',3''-piperidine]-2,4''-dione (II) Geometric parameters (Å, º)

**Table:**

O1—C1	1.218 (2)	C17—H17	0.9300
O2—C24	1.212 (2)	C18—C19	1.385 (2)
N1—C5	1.450 (2)	C18—H18	0.9300
N1—C3	1.453 (2)	C19—C20	1.518 (2)
N1—C4	1.454 (2)	C20—C21	1.529 (3)
N2—C22	1.452 (2)	C20—H20	0.9800
N2—C21	1.456 (2)	C21—H21A	0.9700
N2—C23	1.466 (2)	C21—H21B	0.9700
C1—C6	1.496 (2)	C22—H22A	0.9600
C1—C2	1.534 (2)	C22—H22B	0.9600
C2—C3	1.526 (2)	C22—H22C	0.9600
C2—C20	1.573 (2)	C23—C33	1.525 (2)
C2—C23	1.589 (2)	C23—C24	1.575 (3)
C3—H3A	0.9700	C24—C25	1.469 (3)
C3—H3B	0.9700	C25—C26	1.374 (3)
C4—H4A	0.9600	C25—C34	1.390 (3)
C4—H4B	0.9600	C26—C27	1.390 (3)
C4—H4C	0.9600	C26—H26	0.9300
C5—C6	1.508 (3)	C27—C28	1.363 (3)
C5—H5A	0.9700	C27—H27	0.9300
C5—H5B	0.9700	C28—C29	1.417 (3)
C6—C7	1.331 (2)	C28—H28	0.9300
C7—C8	1.470 (3)	C29—C34	1.398 (3)
C7—H7	0.9300	C29—C30	1.405 (3)
C8—C9	1.384 (3)	C30—C31	1.366 (3)
C8—C13	1.396 (3)	C30—H30	0.9300
C9—C10	1.374 (3)	C31—C32	1.423 (3)
C9—H9	0.9300	C31—H31	0.9300
C10—C11	1.369 (4)	C32—C33	1.360 (2)
C10—H10	0.9300	C32—H32	0.9300
C11—C12	1.358 (4)	C33—C34	1.412 (2)
C11—H11	0.9300	C35—H35A	0.9600
C12—C13	1.392 (3)	C35—H35B	0.9600
C12—H12	0.9300	C35—H35C	0.9600
C13—C35	1.500 (3)	C35—H35D	0.9600
C14—C15	1.390 (3)	C35—H35E	0.9600
C14—C19	1.400 (3)	C35—H35F	0.9600
C14—C36	1.501 (3)	C36—H36A	0.9600
C15—C16	1.368 (3)	C36—H36B	0.9600
C15—H15	0.9300	C36—H36C	0.9600
C16—C17	1.369 (3)	C36—H36D	0.9600
C16—H16	0.9300	C36—H36E	0.9600
C17—C18	1.378 (3)	C36—H36F	0.9600
			
C5—N1—C3	109.05 (14)	C19—C20—C21	116.01 (14)
C5—N1—C4	111.16 (15)	C19—C20—C2	114.95 (14)
C3—N1—C4	112.30 (14)	C21—C20—C2	104.99 (14)
C22—N2—C21	114.57 (15)	C19—C20—H20	106.8
C22—N2—C23	116.08 (15)	C21—C20—H20	106.8
C21—N2—C23	107.05 (13)	C2—C20—H20	106.8
O1—C1—C6	120.91 (16)	N2—C21—C20	103.63 (13)
O1—C1—C2	120.90 (16)	N2—C21—H21A	111.0
C6—C1—C2	118.18 (15)	C20—C21—H21A	111.0
C3—C2—C1	107.64 (14)	N2—C21—H21B	111.0
C3—C2—C20	114.25 (13)	C20—C21—H21B	111.0
C1—C2—C20	111.07 (13)	H21A—C21—H21B	109.0
C3—C2—C23	112.19 (13)	N2—C22—H22A	109.5
C1—C2—C23	107.70 (13)	N2—C22—H22B	109.5
C20—C2—C23	103.83 (13)	H22A—C22—H22B	109.5
N1—C3—C2	108.55 (13)	N2—C22—H22C	109.5
N1—C3—H3A	110.0	H22A—C22—H22C	109.5
C2—C3—H3A	110.0	H22B—C22—H22C	109.5
N1—C3—H3B	110.0	N2—C23—C33	110.81 (14)
C2—C3—H3B	110.0	N2—C23—C24	113.84 (14)
H3A—C3—H3B	108.4	C33—C23—C24	101.21 (14)
N1—C4—H4A	109.5	N2—C23—C2	101.76 (13)
N1—C4—H4B	109.5	C33—C23—C2	119.65 (14)
H4A—C4—H4B	109.5	C24—C23—C2	110.12 (13)
N1—C4—H4C	109.5	O2—C24—C25	126.46 (18)
H4A—C4—H4C	109.5	O2—C24—C23	124.94 (17)
H4B—C4—H4C	109.5	C25—C24—C23	108.48 (16)
N1—C5—C6	112.26 (15)	C26—C25—C34	120.42 (19)
N1—C5—H5A	109.2	C26—C25—C24	132.3 (2)
C6—C5—H5A	109.2	C34—C25—C24	107.31 (16)
N1—C5—H5B	109.2	C25—C26—C27	118.3 (2)
C6—C5—H5B	109.2	C25—C26—H26	120.9
H5A—C5—H5B	107.9	C27—C26—H26	120.9
C7—C6—C1	117.20 (17)	C28—C27—C26	121.8 (2)
C7—C6—C5	123.34 (17)	C28—C27—H27	119.1
C1—C6—C5	119.41 (15)	C26—C27—H27	119.1
C6—C7—C8	128.6 (2)	C27—C28—C29	121.4 (2)
C6—C7—H7	115.7	C27—C28—H28	119.3
C8—C7—H7	115.7	C29—C28—H28	119.3
C9—C8—C13	119.01 (19)	C34—C29—C30	116.11 (19)
C9—C8—C7	121.34 (19)	C34—C29—C28	115.7 (2)
C13—C8—C7	119.6 (2)	C30—C29—C28	128.2 (2)
C10—C9—C8	121.3 (2)	C31—C30—C29	120.30 (19)
C10—C9—H9	119.3	C31—C30—H30	119.8
C8—C9—H9	119.3	C29—C30—H30	119.8
C11—C10—C9	119.8 (3)	C30—C31—C32	122.5 (2)
C11—C10—H10	120.1	C30—C31—H31	118.8
C9—C10—H10	120.1	C32—C31—H31	118.8
C12—C11—C10	119.5 (2)	C33—C32—C31	118.75 (19)
C12—C11—H11	120.3	C33—C32—H32	120.6
C10—C11—H11	120.3	C31—C32—H32	120.6
C11—C12—C13	122.4 (2)	C32—C33—C34	118.17 (17)
C11—C12—H12	118.8	C32—C33—C23	132.64 (16)
C13—C12—H12	118.8	C34—C33—C23	109.07 (15)
C12—C13—C8	118.0 (2)	C25—C34—C29	122.33 (19)
C12—C13—C35	120.5 (2)	C25—C34—C33	113.55 (16)
C8—C13—C35	121.46 (19)	C29—C34—C33	124.10 (19)
C15—C14—C19	118.55 (18)	C13—C35—H35A	109.5
C15—C14—C36	119.47 (18)	C13—C35—H35B	109.5
C19—C14—C36	121.98 (17)	H35A—C35—H35B	109.5
C16—C15—C14	122.1 (2)	C13—C35—H35C	109.5
C16—C15—H15	118.9	H35A—C35—H35C	109.5
C14—C15—H15	118.9	H35B—C35—H35C	109.5
C15—C16—C17	119.4 (2)	H35D—C35—H35E	109.5
C15—C16—H16	120.3	H35D—C35—H35F	109.5
C17—C16—H16	120.3	H35E—C35—H35F	109.5
C16—C17—C18	119.8 (2)	C14—C36—H36A	109.5
C16—C17—H17	120.1	C14—C36—H36B	109.5
C18—C17—H17	120.1	H36A—C36—H36B	109.5
C17—C18—C19	121.8 (2)	C14—C36—H36C	109.5
C17—C18—H18	119.1	H36A—C36—H36C	109.5
C19—C18—H18	119.1	H36B—C36—H36C	109.5
C18—C19—C14	118.42 (17)	H36D—C36—H36E	109.5
C18—C19—C20	121.30 (17)	H36D—C36—H36F	109.5
C14—C19—C20	120.26 (15)	H36E—C36—H36F	109.5
			
O1—C1—C2—C3	−152.18 (17)	C19—C20—C21—N2	−153.51 (14)
C6—C1—C2—C3	29.1 (2)	C2—C20—C21—N2	−25.45 (17)
O1—C1—C2—C20	−26.4 (2)	C22—N2—C23—C33	60.2 (2)
C6—C1—C2—C20	154.89 (15)	C21—N2—C23—C33	−170.39 (14)
O1—C1—C2—C23	86.66 (19)	C22—N2—C23—C24	−53.0 (2)
C6—C1—C2—C23	−92.04 (17)	C21—N2—C23—C24	76.33 (18)
C5—N1—C3—C2	76.18 (17)	C22—N2—C23—C2	−171.47 (15)
C4—N1—C3—C2	−160.14 (15)	C21—N2—C23—C2	−42.10 (16)
C1—C2—C3—N1	−60.49 (17)	C3—C2—C23—N2	147.72 (13)
C20—C2—C3—N1	175.66 (13)	C1—C2—C23—N2	−94.01 (15)
C23—C2—C3—N1	57.82 (17)	C20—C2—C23—N2	23.85 (15)
C3—N1—C5—C6	−54.01 (19)	C3—C2—C23—C33	−89.87 (17)
C4—N1—C5—C6	−178.36 (15)	C1—C2—C23—C33	28.4 (2)
O1—C1—C6—C7	−12.8 (3)	C20—C2—C23—C33	146.26 (14)
C2—C1—C6—C7	165.91 (16)	C3—C2—C23—C24	26.65 (18)
O1—C1—C6—C5	169.68 (17)	C1—C2—C23—C24	144.93 (14)
C2—C1—C6—C5	−11.6 (2)	C20—C2—C23—C24	−97.21 (15)
N1—C5—C6—C7	−154.62 (18)	N2—C23—C24—O2	−51.5 (2)
N1—C5—C6—C1	22.8 (2)	C33—C23—C24—O2	−170.37 (18)
C1—C6—C7—C8	−178.84 (19)	C2—C23—C24—O2	62.1 (2)
C5—C6—C7—C8	−1.4 (3)	N2—C23—C24—C25	124.97 (16)
C6—C7—C8—C9	−46.1 (3)	C33—C23—C24—C25	6.06 (18)
C6—C7—C8—C13	136.6 (2)	C2—C23—C24—C25	−121.50 (15)
C13—C8—C9—C10	−0.4 (4)	O2—C24—C25—C26	−8.7 (4)
C7—C8—C9—C10	−177.7 (3)	C23—C24—C25—C26	174.9 (2)
C8—C9—C10—C11	−0.5 (5)	O2—C24—C25—C34	170.5 (2)
C9—C10—C11—C12	0.6 (6)	C23—C24—C25—C34	−5.9 (2)
C10—C11—C12—C13	0.2 (5)	C34—C25—C26—C27	−0.7 (3)
C11—C12—C13—C8	−1.1 (5)	C24—C25—C26—C27	178.4 (2)
C11—C12—C13—C35	−179.0 (3)	C25—C26—C27—C28	−2.0 (4)
C9—C8—C13—C12	1.2 (4)	C26—C27—C28—C29	1.8 (4)
C7—C8—C13—C12	178.6 (2)	C27—C28—C29—C34	1.1 (3)
C9—C8—C13—C35	179.0 (3)	C27—C28—C29—C30	−178.6 (2)
C7—C8—C13—C35	−3.6 (4)	C34—C29—C30—C31	0.3 (3)
C19—C14—C15—C16	1.2 (3)	C28—C29—C30—C31	−179.9 (2)
C36—C14—C15—C16	−178.30 (19)	C29—C30—C31—C32	1.6 (3)
C14—C15—C16—C17	−0.7 (3)	C30—C31—C32—C33	−1.3 (3)
C15—C16—C17—C18	−0.1 (3)	C31—C32—C33—C34	−1.0 (3)
C16—C17—C18—C19	0.3 (3)	C31—C32—C33—C23	−176.46 (17)
C17—C18—C19—C14	0.3 (3)	N2—C23—C33—C32	50.5 (3)
C17—C18—C19—C20	178.85 (17)	C24—C23—C33—C32	171.57 (19)
C15—C14—C19—C18	−1.0 (3)	C2—C23—C33—C32	−67.4 (2)
C36—C14—C19—C18	178.52 (17)	N2—C23—C33—C34	−125.23 (15)
C15—C14—C19—C20	−179.60 (16)	C24—C23—C33—C34	−4.16 (17)
C36—C14—C19—C20	−0.1 (3)	C2—C23—C33—C34	116.91 (16)
C18—C19—C20—C21	36.9 (2)	C26—C25—C34—C29	3.7 (3)
C14—C19—C20—C21	−144.55 (17)	C24—C25—C34—C29	−175.53 (17)
C18—C19—C20—C2	−86.1 (2)	C26—C25—C34—C33	−177.43 (17)
C14—C19—C20—C2	92.47 (19)	C24—C25—C34—C33	3.3 (2)
C3—C2—C20—C19	6.9 (2)	C30—C29—C34—C25	175.91 (18)
C1—C2—C20—C19	−115.06 (16)	C28—C29—C34—C25	−3.9 (3)
C23—C2—C20—C19	129.45 (15)	C30—C29—C34—C33	−2.8 (3)
C3—C2—C20—C21	−121.77 (16)	C28—C29—C34—C33	177.44 (17)
C1—C2—C20—C21	116.24 (16)	C32—C33—C34—C25	−175.63 (17)
C23—C2—C20—C21	0.75 (16)	C23—C33—C34—C25	0.8 (2)
C22—N2—C21—C20	173.69 (16)	C32—C33—C34—C29	3.2 (3)
C23—N2—C21—C20	43.47 (18)	C23—C33—C34—C29	179.61 (17)

### 1'-Methyl-5''-(2-methylbenzylidene)-4'-(2-methylphenyl)dispiro[acenaphthene-1,2'-pyrrolidine-3',3''-piperidine]-2,4''-dione (II) Hydrogen-bond geometry (Å, º)

*Cg*2 is the centroid of the C8–C13 ring.

**Table:**

*D*—H···*A*	*D*—H	H···*A*	*D*···*A*	*D*—H···*A*
C36—H36*A*···O1	0.96	2.66	3.586 (3)	161
C10—H10···O2^i^	0.93	2.77	3.529 (3)	140
C16—H16···O2^ii^	0.93	2.79	3.530 (3)	137
C35—H35*F*···*Cg*2^iii^	0.96	2.94	3.805 (4)	151

Symmetry codes: (i) −*x*, *y*+1/2, −*z*+1/2; (ii) −*x*, −*y*+2, −*z*; (iii) −*x*+1, −*y*+2, −*z*+1.
